# Supplementary material for: Detection and Evaluation of Blast Resistance Genes in Backbone Indica Rice Varieties from South China
Source: Plants (Basel). 2024 Aug 1;13(15):2134. doi: 10.3390/plants13152134 (PMC11314011; doi:10.3390/plants13152134)
Supplement: Supplementary file 1 [file plants-13-02134-s001.zip › plants-3076723-supplementary.pdf]

Table S1 Varieties information

Number	Rice Germplasm
23P-1	Nei 5B
23P-2	Xiang 2B
23P-3	Xiang B
23P-4	Im
23P-5	Nei 10B
23P-6	Nei 10B/Chuan 106B
23P-7	Xia Dao 1st
23P-8	Zhongzhi 11
23P-9	R227
23P-10	Nongxiang 39
23P-11	Nongxiang 40
23P-12	Nongxiang 42
23P-13	Xiangwan Lian 17th
23P-14	Yan R001
23P-15	Yan R002
23P-16	Jinggan Henxiang Cailiao
23P-17	Ganxiang Zhan
23P-18	Sanjiu Si Miao
23P-19	9311B
23P-20	9311-resistant
23P-21	Fudao 88
23P-22	Yuhe
23P-23	T-8
23P-24	Zhongsizhan 1st
23P-25	Zhongsizhan 4th
23P-26	Zhongsizhan 6th
23P-27	Zhongxiang B
23P-28	Youzhi 3
23P-29	Zhongxiang 1st
23P-30	Zhongzhe B
23P-31	Hejia / Yue Nong Si Miao
23P-32	Guguangzhan / Xin 713
23P-33	Guguangzhan / R1128 // Yue Nong Si Miao
23P-34	H005
23P-35	Qinben Yang
23P-36	Nai Di Ge 9311
23P-37	Xian Ai Zhong Zu 14 Beijing
23P-38	VR73235 Xianai Zhuxi
23P-39	6216 Youzhi
23P-40	Huahang 48
23P-41	Hengxiang Zhan
23P-42	Yu Jing 91

23P-43	Liuzhan
23P-44	Yue Nong Si Miao / Huanghua Zhan
23P-45	Yue Nong Si Miao / Huanghua Zhan
23P-46	Jiuxiang Zhan / Yue Nong Si Miao
23P-47	Lixiang Zhan
23P-48	Xiangxiu Zhan
23P-49	Biao 8
23P-50	Yuehe Si Miao
23P-51	Meixiang Zhan 2nd
23P-52	Taifeng B
23P-53	Hengfeng B
23P-54	Yue Nong Si Miao
23P-55	Hua Zhan
23P-56	Guangchao 1421
23P-57	EF1-1339 3-resistant
23P-58	AC3206
23P-59	R19777
23P-60	Yue Nong Si Miao
23P-61	Wushan Si Miao
23P-62	You Zhan
23P-63	Fumei Zhan
23P-64	Jiuxiang Zhan
23P-65	Hexi Xiang 8th
23P-66	Xinxiang Zhan
23P-67	Teyou Zhan
23P-68	Mei Zhan
23P-69	Guang 8B
23P-70	Biao 24
23P-71	Yue Nong Si Miao /// Jin Nong Si Miao / R8006 / R-resistant 85
23P-72	Yue Nong Si Miao // Jin Nong Si Miao / CNR41
23P-73	Nanjing Xiang Zhan
23P-74	19 Xiang
23P-75	Biao 29
23P-76	Ben 278
23P-77	Da 2
23P-78	Da 4
23P-79	Qingxiang B
23P-80	Da 12
23P-81	Da 22
23P-82	Da 30
23P-83	Da 35
23P-84	Da 39
23P-85	Da 63
23P-86	Jinhang Ruan Zhan

23P-87	Qingxiang B
23P-88	Jinmei Zhan
23P-89	Jinhuang Zhan
23P-90	Jinqing Zhan
23P-91	Yexiang B

Table S2 Markers utilized in this study

Resistant genes	Marker	Primer sequence (5′ –3′)	Annealing temperature (℃)	Expected size (bp)
Pi1	RM224	F: ATCGATCGATCTTCACGAGG<br>R: TGCTATAAAAGGCATTCAAA	55	163
Pi2	AP22	F: GTGCATGAGTCCAGCTCAAA<br>R: GTGTACTCCCATGGCTGCTC	58	143
Pi5	M-Pi5	F:ATAGATCATGCGCCCTCTTG<br>R: TCATACCCCATTCGGTCATT	55	206
Pi9	RM3330	F: CGTTCGAGCAGAACCATCTACC<br>R: CCTCTTCCGCTCCACTCTCC	58	170
Pia	PIA	F: GCGACTGACACTTTCAATAGC<br>R: CGGTAGAGCAATTTAGAAGCAG	55	189
Pid2	RM527	F: GGCTCGATCTAGAAAATCCG<br>R: TGCACAGGTTGCGATAGAG	55	233
Pid3	Primer9	F: TGCCGAGAGGAGGATTAGGT<br>R: CAGGTAAGAGGTGGTTT	55	430
Pigm	ZJ58.7	F: ACTTGCTGGGAGAAGGATT<br>R: AGTTCGTACTTTTCAGGCT	55	236
pikh	Primer10	F:TGGATGACAAGAACCGAGCC<br>R: GTGAGAGAGGAAAGATGCTGC	55	563
Pita	YL155/187	F:AGCAGGTTATAAGCTAGGCC<br>R: CTACCAACAAGTTCATCAAA	58	1042
